# Annotations

**Published:** 1904-06-04

**Authors:** 


### ANNOTATIONS.

### Professional Etiquette.

A WRITER in one of the daily papers who has constituted herself a censor of morals and has taken pains to advertise her selected occupation, has recently been presenting in somewhat lurid tints a picture of the disastrous results which are produced by the adherence of the medical profession to certain rules of conduct. Numbers of "human lives," it appears, are sacrificed to medical etiquette. The canons of this code must be satisfied "even if the murdered sufferer's friends are not." This same etiquette is also, we are told, a staunch foe to medical progress and provides restrictions of such a character that the "merest hint" of any new discovery or invention is condemned. No doubt all this is in its way excellent copy, and well calculated to make the flesh creep. To take it seriously would be absurd, Intelligent people, of course, readily appreciate the value of the ethical rules which govern the conduct of medical men in their mutual professional relations and in their relations to the public and understand that such rules are framed, not for selfish purposes, but in the public interest. Still it must be admitted that there are large numbers of people who believe that medical men are under the discipline of an elaborate and artificial code which prescribes their conduct in various circumstances and which they disobey at their peril. Hence it is well for practitioners to be prepared to explain that this is largely a delusion, and that no man's proper liberty is restricted other than to the public advantage. There is certainly no rule or regulation inconsistent with the propositions that the welfare of the patient is the first consideration, and that no man should do to his neighbour other than he would have his neighbour do to himself. The reports of our professional societies and the columns of the medical press show how far removed from accuracy is the statement that new suggestions and inventions are hampered and condemned by the "medical college and its noble body of autocrats."

### Pharmacy on the Continent.

IN a recent issue of the *Pharmaceutical Journal*, a contributor gives an interesting account of his experiences as a pharmacist in several Continental cities. He notes that the Continental prescription as a rule is much more simple in its composition than the English one and often consists of one or two ingredients only. Hypodermic injections he found to be largely ordered and to be dispensed always as sterilised solutions. Generally the art of the pharmacist is directed to the production of preparations which will command the confidence and support of the medical profession, and is little concerned with compounds which can be presented to the public as nostrums. The writer of the paper is disposed to attribute this to the fact that medical men are prevented by law from dispensing medicines, and thus a relationship is established between physician and pharmacist which increases the professional and social status of the latter. The law, as is well known, with equal measure restricts the pharmacist to his legitimate business and prevents him from giving medical advice or recommending particular remedies in the treatment of disease. Whether these rules could or could not be adopted with advantage in this country is a moot point, but it is practically certain that here they would have to be the outcome of a voluntary decision and arrangement and not the imperative orders of legal enactment. One practice common in France might well be transferred to this country, namely, the rule according to which a prescription is not dispensed a second time without the consent and authority of the medical attendant. At Continental pharmacies, it seems, a considerable amount of work is done in chemical and bacteriological examinations which at home would be referred to a clinical laboratory. The three years' curriculum through which pharmacists of the first rank have to pass includes a thorough training in these investigations. Altogether, the writer of the paper now in question conveys the impression that the foreign pharmacist is both more highly trained and is engaged in a higher class of work than is the case with his British *confrères.*

### Women and Whisky.

A LADY, writing in the *Graphic*, makes, in a casual sort of manner, an unpleasant charge against the members of the medical profession. Having mentioned with approval a report that the German Emperor had become a teetotaller, and recorded her impression that there is in England a distinct diminution in the ordinary drinking of wine and whisky at lunch and at dinner, she goes on to assert that "it is the doctors who have brought women to drink whisky by constantly recommending it to their patients, and in so doing have brought many of them to ruin." We do not believe that the charge itself is true. That medical men occasionally consider it necessary to recommend a patient to take a small quantity of whisky and water with a meal is probable. Whisky as an alternative to wine may undoubtedly be employed medicinally with advantage in certain cases, both for men and for women. But this is very different from the constant recommendation which is suggested, as if, indeed, doctors regard whisky as a sort of panacea for every disease under the sun, and take a perfect delight in urging its consumption upon their patients. As a matter of fact, there never was a time when medical men were more slow to prescribe the use of alcohol in any form than they are in the present day; nor a time when so many refrained from advising its use at all. Even if, however, it were the practice of the profession to "constantly recommend" women to take a small quantity of whisky with their food for the benefit of their health, we deny that anyone would be justified in ascribing to them the ruin of their patients. It is the primary duty of the physician to do his best to cure the person for whom he prescribes, and if, with that object in view, he advises the restricted employment of a stimulant or a drug, he is not to blame if his patient subsequently uses it without restriction. Adults of sane mind are accountable for their own actions, and we protest against the growing habit of saddling other persons with responsibility for their misdeeds on the slightest possible pretext. Women who drink whisky in excess cannot for a moment be permitted to excuse themselves by advancing the utterly absurd and futile plea that "the doctor recommended its use."

